# Supplementary figures and images for: Molecular and structural reprogramming of gastric cancer revealed by systems-level transcriptomic analysis
Source: PLoS One. 2026 May 21;21(5):e0344143. doi: 10.1371/journal.pone.0344143 (PMC13193547; doi:10.1371/journal.pone.0344143)

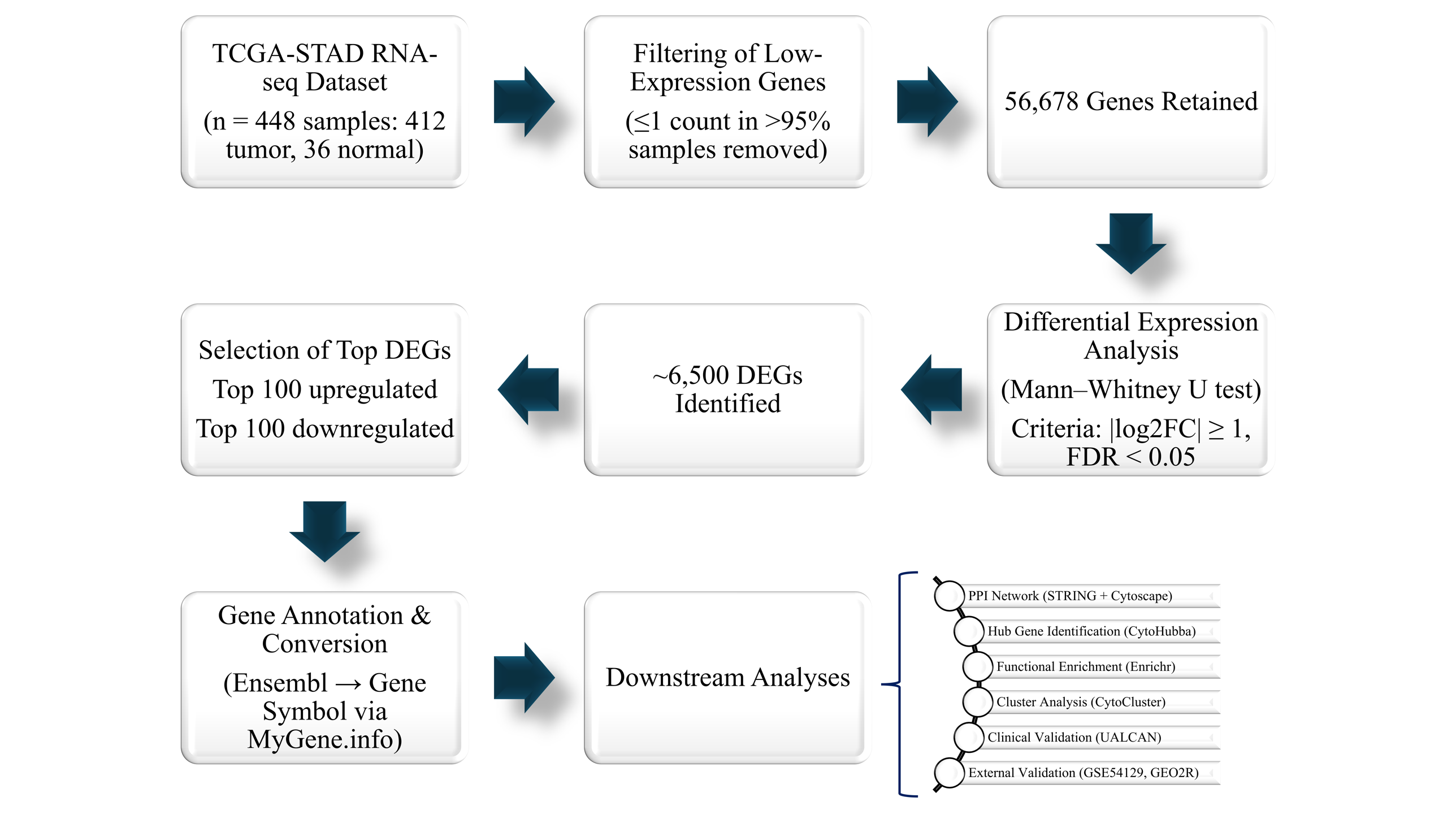

Supplement: S1 Fig — This figure includes the data acquisition from TCGA-STAD, preprocessing, differential expression analysis, functional enrichment, PPI network construction, hub gene identification, clustering, and external validation. (TIF) [file pone.0344143.s001.tif]
